# Quantitative and Qualitative Analysis of Circulating Cell-Free DNA Can Be Used as an Adjuvant Tool for Prostate Cancer Screening: A Meta-Analysis

**DOI:** 10.1155/2016/3825819

**Published:** 2016-09-27

**Authors:** Changqing Yin, Changliang Luo, Wei Hu, Xu Ding, Chunhui Yuan, Fubing Wang

**Affiliations:** ^1^Department of Laboratory Medicine and Center for Gene Diagnosis, Zhongnan Hospital of Wuhan University, Wuhan, China; ^2^Department of Laboratory Medicine, The First People's Hospital of Jingmen, Jingmen, China; ^3^Department of Immunology, School of Basic Medical Sciences, Wuhan University, Wuhan, China

## Abstract

As part of “liquid biopsy,” lots of literature indicated the potential diagnostic value of circulating cell-free DNA (cfDNA) in the management of prostate cancer (PCa). However, the literature on the accuracy of cfDNA detection in PCa has been inconsistent. Hence, we performed this meta-analysis to assess the diagnostic value of cfDNA in PCa. A total of 19 articles were included in this analysis according to the inclusion and exclusion criteria. We then investigated two main subgroups in this meta-analysis, including qualitative analysis of abnormal level of cfDNA and qualitative analysis of single-gene methylation alterations. Overall, the results of quantitative analysis showed sensitivity of 0.73 (95% CI, 0.62–0.82) and specificity of 0.80 (95% CI, 0.70–0.87), with an area under the curve (AUC) of 0.83 (95% CI, 0.80–0.86). For qualitative assessment, the values were 0.34 (95% CI, 0.22–0.48), 0.99 (95% CI, 0.97–1.00), and 0.91 (95% CI, 0.88–0.93), respectively. Our results suggest the pooled specificity of each subgroup is much higher than the specificity of prostate-specific antigen (PSA). However, they are not recommended for PCa screening alone, because their sensitivities are not higher than the conventional serum biomarkers PSA. We conclude that analysis of cfDNA can be used as an adjuvant tool for PCa screening.

## 1. Introduction

Prostate cancer (PCa) is the second most frequently diagnosed noncutaneous cancer in males worldwide [1]. In the United States, it gradually tends to be the second leading cause of cancer death, accounting for approximately 220,800 new patients and 27,450 deaths in 2015 [1]. As with other cancers, if PCa patients could be diagnosed at its early stage, the treatment success rate would be greatly improved. Currently, early detection of PCa is still predominantly based on serum PSA levels, transrectal ultrasonography (TRUS), and digital rectal exam (DRE) [2–4]. Among them, PSA test is used as “gold standard” for PCa screening. However, despite its relatively higher sensitivity, PSA test sill results in a great number of avoidable prostate biopsies and increased healthcare costs due to the low specificity [5]. More seriously, patients with high-grade PCa may even have normal PSA levels according to the PSA test [6]. Hence, there is an urgent need for novel markers that can either outperform the conventional biomarkers or be used as adjuvant for PSA to complement the poor specificity in managing PCa patients.

Recently, the presence of cfDNA has been highlighted in its diagnostic value and management of human cancers as an integral part of “liquid biopsy.” Analysis of cfDNA has recently been recognized as a minimally invasive method to explore tissue characteristics. It is presumed that cfDNA (150–200 nucleotides in length) is usually derived from normal or tumor cells through apoptosis or necrosis [7, 8], which mainly are composed of tumorous cfDNA in the cancer patient [9]. The abnormalities of circulating cfDNA include both quantitative and qualitative changes. Quantitative abnormalities contribute to the aberrant levels of cfDNA, while qualitative changes include single-gene methylation alterations and mutations, DNA integrity, loss of heterozygosity (LOH), and circulating nucleosomes.

A high number of studies have indicated that PCa patients have higher cfDNA concentrations than controls [10–16]. In addition, DNA methylation in tumor suppressor gene is a frequent epigenetic modification in human malignancies. PCa is not an exception. Many articles have reported hypermethylated promoters in cfDNA, including GADD45a [10], GSTP1 [17], CDH13 [18], MDR1 [19, 20], RASSF1 [17, 19], APC [19, 21], PTGS2 [13, 19], Reprimo [13], RARB2 [17, 21], and TIG1 [13]. Among them, hypermethylated GSTP1 has been proved to be the most frequently observed somatic genome alteration in PCa, with a relatively high specificity [17]. All of the above studies have indicated the potential value of cfDNA as a novel biomarker for PCa screening; however, inconsistent conclusions still exist in the literature due to differences in ethnicity, assay methods, sample types, source of controls, and methylation gene location. Hence, this meta-analysis was performed to comprehensively elucidate the diagnostic value of cfDNA for PCa screening.

## 2. Materials and Methods

### 2.1. Data Sources and Search

We conducted this meta-analysis under the guidelines of the Preferred Reporting Items for Systematic Reviews and Meta-Analyses (PRISMA). To retrieve all eligible articles, PubMed and Embase databases and Cochrane Library and Chinese National Knowledge Infrastructure (CNKI) were comprehensively searched up to 1 May 2016 without language limitation. The search medical subject heading (MeSH) terms employed for literature retrieval included “prostate cancer” or “prostate neoplasm”; “cell free DNA” or “cfDNA” or “circulating DNA” or “plasma/serum DNA”; and “diagnosis” or “sensitivity” or “specificity” or “accuracy”. The reference lists of eligible articles were also independently searched to obtain other valuable sources.

### 2.2. Study Selection Criteria

To be qualified for inclusion in this meta-analysis, articles must comply with all the following criteria: (1) articles evaluated the diagnostic value of cfDNA in plasma/serum or blood for PCa; (2) patients had confirmed PCa by a gold standard test; and (3) articles provided sufficient data (e.g., sensitivity [SEN], specificity [SPE], true positives [TP], false positives [FP], false negatives [FN], and true negatives [TN]). Meanwhile the major exclusion criteria were as follows: (1) studies with duplicate data reported in other studies and (2) reviews, technical reports, case reports, comments, or letters with invalid data.

### 2.3. Data Extraction and Quality Assessment

Two investigators independently reviewed all of the articles and extracted data from the selected articles: first author's name, publication year, characteristics of participants (ethnicity, mean/median age, source of control, number of cases and controls, sample types), assay methods, assay indicators, sensitivity, specificity, and quality assessment information. In addition, based on the revised quality assessment of diagnostic accuracy studies-2 (QUADAS-2) criteria, the included articles were scored independently by four key domains: patient selection, index test, reference standard, and flow and timing [22]. Every quality index has a maximum score value of 14 in each of the studies.

### 2.4. Statistical Analysis

We used the STATA software 14.0 (STATA Corp., College Station, TX, USA) to conduct this meta-analysis. The pooled SEN (TP/[TP + FN]), SPE (TN/[TN + FP]), negative likelihood ratio (NLR), positive likelihood ratios (PLR), and diagnostic odds ratio (DOR) with the 95% confidence intervals (95% CIs) were calculated using the bivariate meta-analysis model [23]. At the same time, we constructed the summary receiver operator characteristic (SROC) curve and calculated the area under the SROC curve based on the sensitivity and specificity of each selected study [24], which can serve as an indicator for the probability of correctly identifying patients from the control. *Q* test and *I* ^2^ statistics were carried out to explore the heterogeneity among studies. *p* value ≤0.10 for *Q* test or *I* ^2^ value ≥50% represented substantial between-study heterogeneity, and then we had to use the random-effects model [25]. In addition, based on the characteristics of the included articles, metaregressions were performed to explore the sources of heterogeneity if necessary. Furthermore, potential presence of public bias was assessed by Deeks' test, with *p* < 0.05 indicating statistical significance.

## 3. Results

### 3.1. Search Results

**Figure 1:**
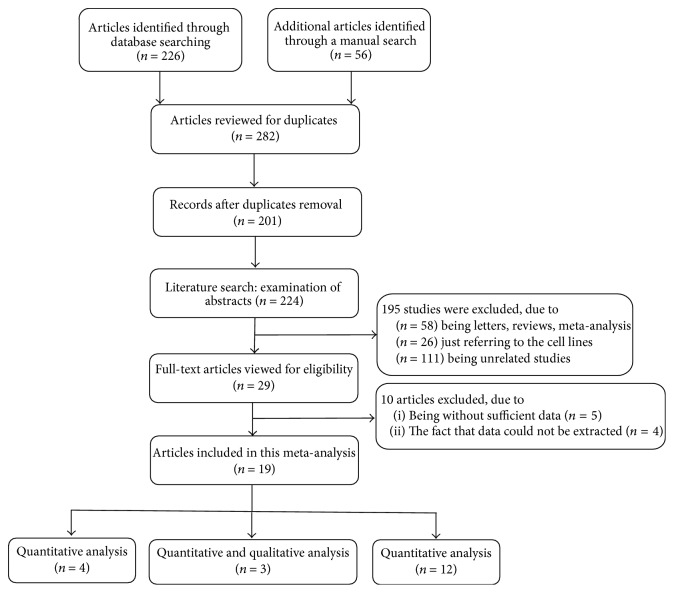
The flow chart of the study selection process in this meta-analysis.

Figure 1 presents the procedure and results of the literature retrieval. After independent review, 29 articles dealing with the quantitative and qualitative analysis of cfDNA for the diagnosis of PCa were selected in the analysis. 10 articles were further excluded due to lacking sufficient data or the fact that the data cannot be extracted by reviewing the full text. Ultimately, a total of 19 articles [10–21, 26–32] were included in the final meta-analysis.

Table 1, in an order by the publication year, summarizes main characteristics of the 19 included articles. All of these selected studies, with the publication years ranging from 2001 to 2015, with 2239 subjects in total, included 1467 PCa. The 772 people without PCa served as control groups, which were mainly composed of healthy volunteers, or benign prostatic hyperplasia (BPH) patients or negative-biopsy patients. Among these 19 articles, 4 articles [11–13, 16] only evaluated abnormal levels of cfDNA in plasma/serum/blood (quantitative analysis group) and 12 articles [17–21, 26–32] assessed single-gene methylation alterations (qualitative analysis group), while 3 articles [10, 14, 15] conducted quantitative and qualitative analysis simultaneously. In addition, most of the subjects were from Europe, with the remaining patients from Asia, North America, and South America. As for the specimen types, serum specimens were included in 6 articles, plasma specimens were included in 10 articles, and blood specimens were included in 2 articles, and 1 article included 2 specimen types.

### 3.2. Diagnostic Accuracy of Quantitative and Qualitative Analysis of cfDNA for the Diagnosis of PCa

**Figure 2:**
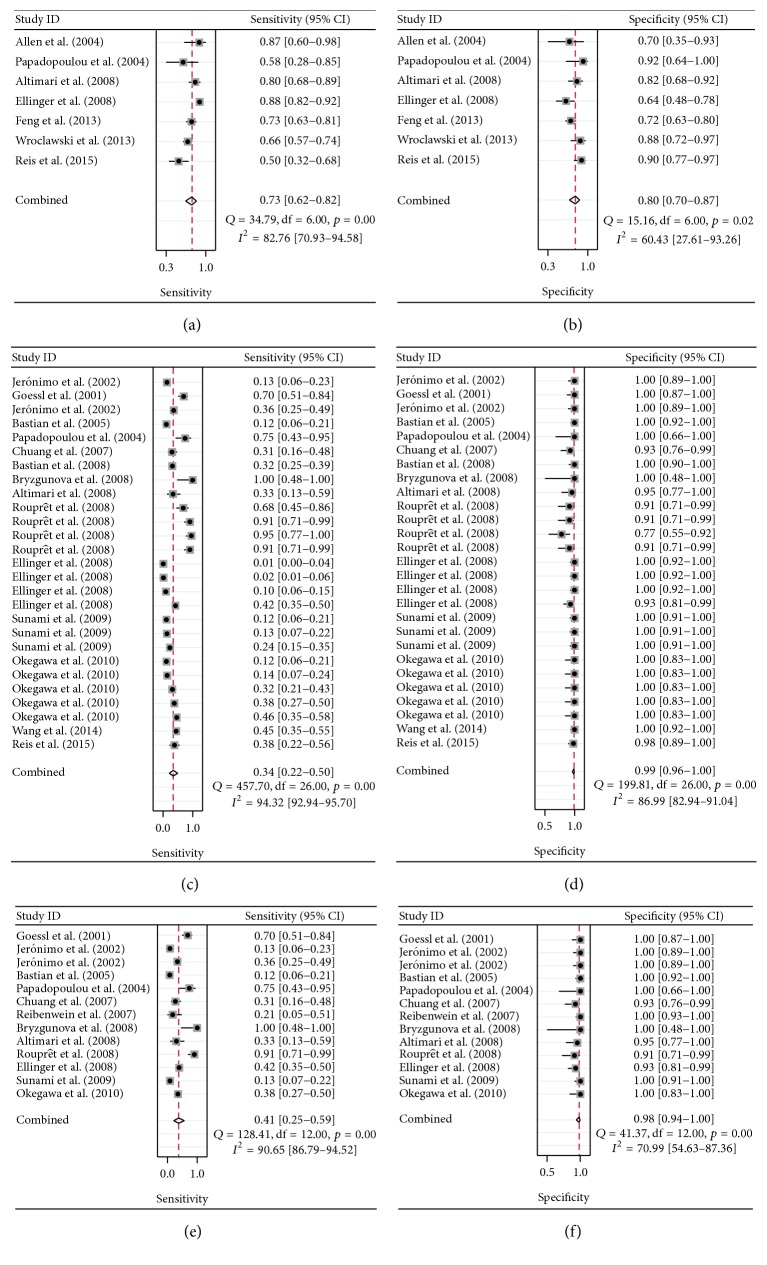
Summary estimates of sensitivity and specificity for the different subgroups with forest plots analysis. (a and b) Forest plots for the quantitative analysis subgroup. (c and d) Forest plots for the qualitative analysis subgroup. (e and f) Forest plots for the GSTP1 hypermethylation analysis subgroup.

**Figure 3:**
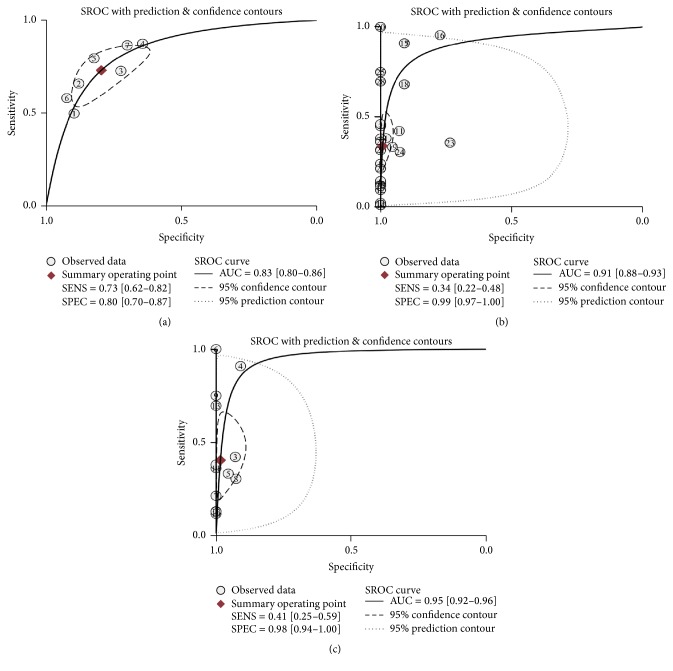
SROC analysis of the diagnostic performance for the different subgroups. (a) SROC curves for the subgroup of quantitative analysis; (b) SROC curves for the subgroup of qualitative analysis; and (c) SROC curves for the GSTP1 hypermethylation analysis subgroup.

The overall pooled SEN and SPE in the quantitative analysis group were 0.73 (95% CI, 0.62–0.82) and 0.80 (95% CI, 0.70–0.87), respectively, for distinguishing patients with PCa from controls (Figures 2(a) and 2(b)). Our results show that PLR was 3.60 (95% CI, 2.60–5.00), NLR was 0.34 (95% CI, 0.25–0.45), and DOR was 11.00 (95% CI, 7.00–16.00). While between-study heterogeneity was significant in the sensitivity and specificity data (*I* ^2^ = 82.76 and *I* ^2^ = 60.43, resp.), thus we calculate the pool estimates in this analysis by using the random-effects model.  Figure 3(a) shows the corresponding SROC curve with AUC of 0.83 (95% CI, 0.80–0.86), indicating that quantitative analysis of cfDNA is capable of differentiating PCa from controls with a relatively high accuracy.

The overall pooled sensitivity and specificity for the qualitative analysis group were 0.34 (95% CI, 0.22–0.48) and 0.99 (95% CI, 0.97–1.00), respectively (Figures 2(c) and 2(d)). The PLR, NLR, and DOR were 43.20 (95% CI, 11.90–156.5), 0.67 (95% CI, 0.55–0.81), and 65.00 (95% CI, 18.00–234.00), respectively. The corresponding overall SROC curves are shown in Figure 3(b), with AUC of 0.91 (95% CI, 0.88–0.93).

### 3.3. Diagnostic Efficiency of Circulating GSTP1 Methylation in PCa

In the studies related to the diagnostic efficacy of qualitative analysis of cfDNA, circulating GSTP1 promoter methylation is the most commonly examined somatic genome alteration in all the selected articles. Therefore, we further analyzed the diagnostic role of GSTP1 methylation in distinguishing PCa patients from controls. In the 12 articles related to GSTP1, the pooled sensitivity was 0.41 (95% CI, 0.25–0.59) and the specificity was 0.98 (95% CI, 0.94–1.00) (Figures 2(e) and 2(f)). The PLR was 24.60 (95% CI, 6.60–92.50), the NLR was 0.60 (95% CI, 0.45–0.81), and the DOR was 41.00 (95% CI, 10.00–164.00). The AUC for GSTP1 was 0.95 (95% CI, 0.92–0.96) (Figure 3(c)).

### 3.4. Subgroup Analysis

Subgroup analyses based on ethnicity, sample types, source of control, assay methods, and methylation gene location were also conducted. As for quantitative analysis of cfDNA, the pooled estimates were both similar in the subgroup of ethnicity and sample types (Table 2). However, the subgroup analysis based on source of control suggested that using healthy controls had higher sensitivity and specificity compared with those using BPH/benign patients with sensitivity of 0.79 versus 0.75 and specificity of 0.82 versus 0.77, respectively. Furthermore, for the qualitative analysis of cfDNA, the pooled sensitivity of each subgroup was lower than 0.5, while the pooled specificity was higher than 0.95, suggesting that the analysis of cfDNA can complement PSA screening for PCa diagnosis regardless of ethnicity, specimen, source of control, assay methods, or methylation gene location.

### 3.5. Heterogeneity and Metaregression Analysis

**Figure 4:**
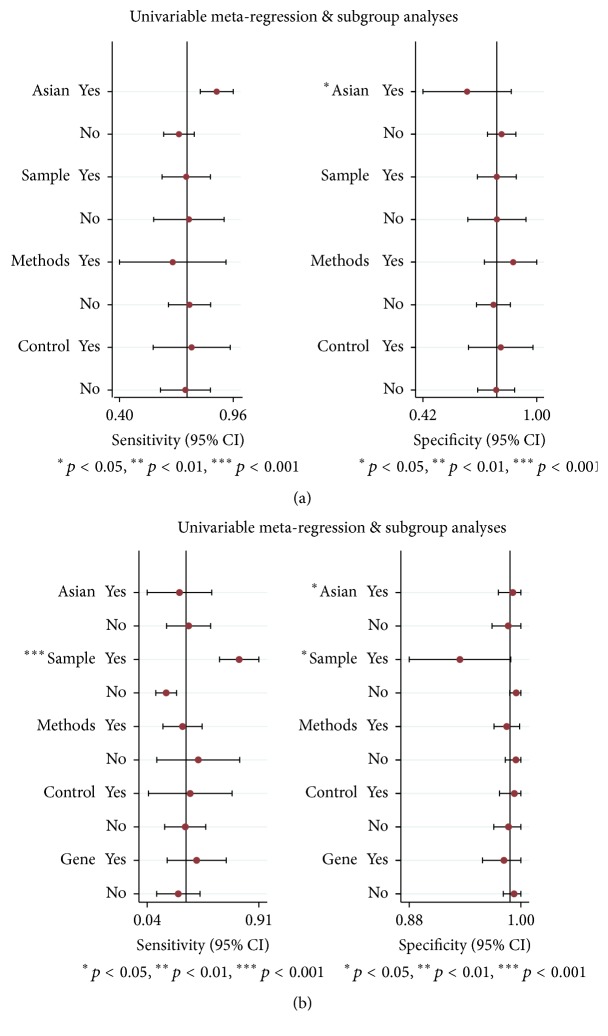
Forest plots of multivariable metaregression and subgroup analyses for SEN and SPE in the subgroup of quantitative analysis (a) and qualitative analysis (b).

The metaregressions were also performed to further explore potential sources of heterogeneity (Figure 4). Our metaregression analysis characteristics included “ethnicity (Asian or not)” and “sample type (Plasma or not)” and “assay methods” and “methylation gene location (gene)”; the metaregression results suggested that the “ethnicity” covariate might be responsible for the heterogeneity in the specificity of both quantitative and qualitative analysis group, and the covariate of “sample types” might produce major heterogeneity in sensitivity of the qualitative analysis group.

### 3.6. Publication Bias

**Figure 5:**
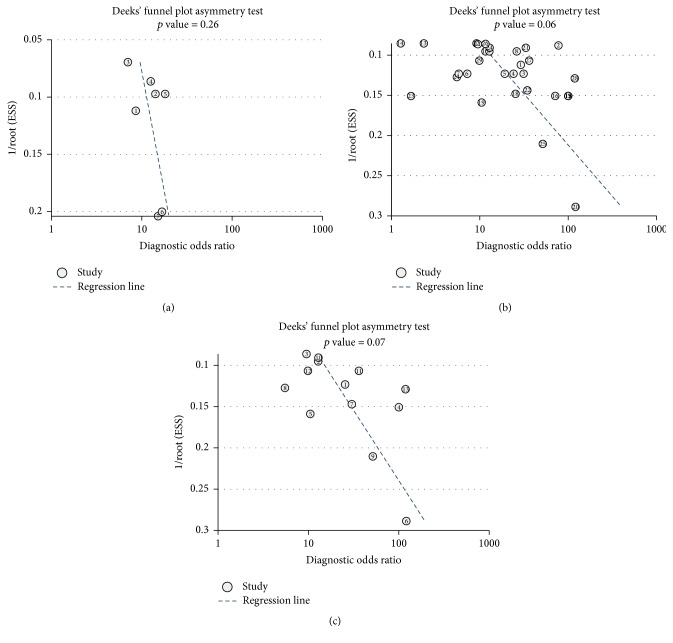
Deeks' test for the assessment of potential publication bias in the different subgroups. (a) Deeks' test for the subgroup of quantitative analysis. (b) Deeks' test for the subgroup of qualitative analysis. (c) Deeks' test for the subgroup of GSTP1 hypermethylation analysis.

Deeks' test was used to assess the potential publication bias of included studies. The slope coefficient was associated with *p* value of 0.26 in the subgroup of quantitative analysis (Figure 5(a)), with *p* value of 0.06 in the subgroup of qualitative analysis (Figure 5(b)), and with *p* value of 0.07 in the GSTP1 methylation subgroup (Figure 5(c)), indicating no significant publication bias existing in our meta-analysis.

## 4. Discussion

Although great achievements have been made in the diagnostic techniques, the currently available biomarkers and imaging assessments give only an adequate performance in the early detection of PCa. Thus, novel molecular markers that can help in early diagnosis are still urgently needed. Numerous studies have demonstrated the potent utility of circulating cancer byproducts detection, namely, “liquid biopsy,” which could provide accessible, accurate, and dynamic information to evaluate tumor progression [33, 34]. In terms of liquid biopsy, cfDNA have been the most studied due to their benefits of easier collection and analysis. The detection of cfDNA can be categorized as quantitative and qualitative analysis. The former one encompasses the isolation and measurement of cfDNA concentration in blood samples in particular, while qualitative approaches include the detection of cfDNA methylation, allelic imbalance, and loss of heterogeneity. Lots of researches have reported the wide range of DNA concentrations in the plasma of PCa and BPH patients [35, 36]. As the analytic methods for the detection of hypermethylated cfDNA in cancer patients are well-established [37], thus, in the qualitative analysis subgroup, we mainly focused on the detection of cfDNA methylation. Wu et al. had conducted a meta-analysis and concluded that GSTP1 promoter methylation measured in plasma, serum, or urine samples, in combination with PSA screening, would significantly enhance the diagnosis accuracy for PCa [38]. In addition, GADD45a [10], RASSF1 [17, 19], and APC [19, 21] methylation have also been frequently studied in the cfDNA of PCa. Bastian et al. even suggested that a combination of multiple DNA methylation markers can capture high sensitivity and specificity compared to the single ones [20]. However, whether cfDNA can be used as a diagnostic marker in PCa patients has not yet been validated owing to varied studies design and results. Therefore, we reviewed the articles about the diagnostic accuracy of quantitative and qualitative analysis of cfDNA in distinguishing patients with PCa from controls and performed a detailed meta-analysis.

The pooled specificity values in each subgroup of quantitative analysis, qualitative analysis, and GSTP1 were 0.80 (95% CI, 0.70–0.87), 0.99 (95% CI, 0.97–1.00), and 0.98 (95% CI, 0.94–1.00) respectively, which were all much higher than the specificity of PSA. PCa diagnostic sensitivity in the quantitative analysis was superior to the subgroup of qualitative analysis or GSTP1, which might be caused by different genetic loci and assay methods. Despite the type of diagnostic indicator chosen, the sensitivity of each subgroup was still not significantly higher than the sensitivity of PSA screening. These results suggest that neither cfDNA nor PSA test is sufficiently accurate for PCa screening. Numerous studies have already indicated that combining cfDNA analysis with PSA for diagnosing and assessing PCa can yield much higher accuracy than does either method on its own. For instance, the combination of PSA with cfDNA concentration (cfDNA ≥ 188 ng/mL) had a specificity of 89.5% and resulted in improvement of sensitivity from 38.2% to 76.5% [10]. In addition, when the GADD45a methylation was involved in the combination, they even yielded AUC of 0.937, with specificity of 87.5% and sensitivity of 94.1% [10]. Chun et al. also found out increased DNA concentration in PCa patients and that the predictive value remarkably increased from 5.6% to 78.3% based on a multivariate model (including total PSA, free/total PSA, and cfDNA) [39]. The encouraging results of our meta-analysis not only validate previous researches in the further step but also support the application of cfDNA-based detection of liquid biopsies for the diagnosis of PCa.

In order to find out the potential sources of heterogeneity, we further carried out subgroup analyses based on ethnicity, sample types, source of control, assay methods, and methylation gene location. The specificity of each subgroup in the qualitative analysis is similar and stable regardless of ethnicity, specimen, source of control, assay methods, or methylation gene location, while, in the quantitative analysis, the detection of cfDNA shows a superior performance in differentiating PCa from healthy controls. Furthermore, we performed a metaregression analysis and the results show that the sources of heterogeneity mainly resulted from differences in ethnicity and sample types.

It should be emphasized that there are still several limitations during the process of our meta-analysis even if we made every effort to limit the bias. Firstly, we may miss several valuable studies during our literature search in spite of the comprehensive search strategy. Furthermore, there are several problems in the eligible articles, such as having different PCR protocols for each of the target genes or the randomly selected healthy controls and BPH controls.

In conclusion, the present results confirmed the strong diagnostic value of cfDNA in PCa. Quantitative and qualitative analysis of cfDNA can be used as an effective adjuvant tool to complement serum PSA assay for the early diagnosis of PCa. In the future, large validation studies are still necessary to clarify the value of cfDNA assay combined with PSA for PCa detection.

## Figures and Tables

**Table 1:** Characteristics and quality assessment of diagnostic clinical trials included in the meta-analysis.

Included studies	Country	Case		Control		Control type	Sample type	Cutoff	Assay methods	Assay indicators	Sensitivity (%)	Specificity (%)	Score
		*N*	Mean age	*N*	Mean age								
Reis et al. (2015) [10]	America	34	64.9	48	64.5	Benign patient	Serum	188 ng/mL	qRT-PCR	Quantitative analysis	50.00%	89.60%	10

Wroclawski et al. (2013) [11]	Brazil	133	66.8	33	64.6	Negative biopsies	Plasma	140 ng/mL	SA	Quantitative analysis	66.00%	88.00%	8

Feng et al. (2013) [12]	China	96	63.2	112	60.3	BPH	Plasma	10 ng/mL	qRT-PCR	Quantitative analysis	73.20%	72.70%	7

Ellinger et al. (2008) [13]	Germany	168	65.8	42	68.8	BPH	Serum	19.7 ng/mL	qRT-PCR	Quantitative analysis	87.50%	64.00%	9

Altimari et al. (2008) [14]	Italy	64	64.5	45	na	Healthy volunteer	Plasma	8 ng/mL	qRT-PCR	Quantitative analysis	80.00%	82.00%	9

Papadopoulou et al. (2004) [15]	Greece	12	na	13	na	Healthy volunteer	Plasma	10 ng/mL	qRT-PCR	Quantitative analysis	58.00%	92.00%	7

Allen et al. (2004) [16]	England	15	68	10	67	Healthy volunteer	Plasma	1000 GE/mL	qRT-PCR	Quantitative analysis	85.00%	73.00%	8

Reis et al. (2015) [10]	America	34	64.9	48	64.5	Benign patient	Serum	89.6^*∗*^	Pyrosequencing (quantitative)	Methylation (GADD45a)	38.20%	97.90%	10

Wang et al. (2014) [18]	China	98	na	47	na	BPH/bladder stone/healthy volunteer	Serum	na	MS-PCR (nonquantitative)	Methylation (CDH13)	44.70%	100.00%	6

Okegawa et al. (2010) [19]	Japan	76	72.2	20	69.4	Benign patient	Serum	na	MS-PCR (quantitative)	Methylation (MDR1)	46.00%	100.00%	8
	—	—	—	—	—			—	—	Methylation (GSTP1)	22.00%	100.00%	—
	—	—	—	—	—			—	—	Methylation (RASSF1A)	18.00%	100.00%	—
	—	—	—	—	—			—	—	Methylation (APC)	15.00%	100.00%	—
	—	—	—	—	—			—	—	Methylation (PTGS2)	12.00%	100.00%	—

Sunami et al. (2009) [17]	Canada	83	70.4	40	na	Healthy volunteer	Serum	na	MS-PCR (quantitative)	Methylation (RASSF1)	24.00%	100.00%	10
	—	—	—	—	—			—	—	Methylation (GSTP1)	13.00%	100.00%	—
	—	—	—	—	—			—	—	Methylation (RARB2)	12.00%	100.00%	—

Ellinger et al. (2008) [32]	Germany	168	65.8	42	68.9	BPH	Serum	na	qMS-PCR (quantitative)	Methylation (GSTP1)	42.30%	92.30%	10
	—	—	—	—	—			—	—	Methylation (TIG1)	9.50%	100.00%	—
	—	—	—	—	—			—	—	Methylation (PTGS2)	2.40%	100.00%	—
	—	—	—	—	—			—	—	Methylation (Reprimo)	1.20%	100.00%	—

Rouprêt et al. (2008) [21]	France	22	73	22	62	BPH	Blood	na	qMS-PCR (quantitative)	Methylation (GSTP1)	91.00%	91.00%	11
	—	—	—	—	—			—	—	Methylation (APC)	91.00%	91.00%	—
	—	—	—	—	—			—	—	Methylation (RAR*β*)	68.00%	91.00%	—

Altimari et al. (2008) [14]	Italy	18	na	22	na	Healthy volunteer	Plasma	na	MS-PCR (nonquantitative)	Methylation (GSTP1)	33.00%	95.00%	

Bryzgunova et al. (2008) [26]	Russia	5	na	5	na	Healthy volunteer	Plasma	na	BS (quantitative)	Methylation (GSTP1)	100.00%	100.00%	

Bastian et al. (2008) [20]	Germany	192	58.9	35	60.1	Negative biopsies	Serum	na	qMS-PCR (quantitative)	Methylation (MDR1)	32.00%	100.00%	10

Reibenwein et al. (2007) [27]	Austria	14	70 (median)	49	na	Healthy volunteer	Serum	na	MS-PCR (nonquantitative)	Methylation (GSTP1)	21.40%	100.00%	12
	—	—	—	—	—	—	—	—	—	Methylation (AR)	40.30%	73.50%	—

Chuang et al. (2007) [28]	Taiwan	36	na	27	na	BPH	Plasma	na	qMS-PCR (quantitative)	Methylation (GSTP1)	31.00%	93.00%	8

Papadopoulou et al. (2004) [15]	Greece	12^&^	na	9	na	Healthy volunteer	Plasma	na	MS-PCR (nonquantitative)	Methylation (GSTP1)	75.00%	100.00%	7

Sunami et al. (2009) [17]	America	85	60.2	46	58.6	Negative biopsies	Serum	na	qMS-PCR (quantitative)	Methylation (GSTP1)	12.00%	100.00%	10

Jerónimo et al. (2002) [29]	America	69	63 (median)	31	64 (median)	BPH	Serum	na	MS-PCR (nonquantitative)	Methylation (GSTP1)	36.00%	100.00%	9
	—	—	—	—	—			—	qMSP (quantitative)	Methylation (GSTP1)	13.00%	100.00%	—

Goessl et al. (2001) [31]	Germany	33	66	26	64	BPH	Plasma/serum	na	MS-PCR (nonquantitative)	Methylation (GSTP1)	72.00%	100.00%	7

BPH = benign prostatic hyperplasia; MS-PCR = methylation-specific PCR; qMS-PCR = quantitative methylation-sensitive PCR; qMSP = quantitative methylation-specific PCR; SA = spectrophotometric assay; GE = genome equivalents, and BS = bisulphite sequencing.

^&^19 patients under therapy were not included.

^*∗*^Methylation index.

**Table 2:** Summary diagnostic performance of miRNAs for prostate cancer.

Analysis	Group	Subgroup	Sensitivity (95% CI)	Specificity (95% CI)
Quantitative analysis	Overall		0.73 (0.62–0.82)	0.80 (0.70–0.87)
	Ethnicity	Asian	0.73 (0.63–0.81)	0.82 (0.63–0.80)
		Other ethnicities	0.74 (0.61–0.84)	0.83 (0.72–0.90)
	Sample types	Serum	0.81 (0.75–0.86)	0.78 (0.68–0.86)
		Plasma	0.78 (0.65–0.78)	0.80 (0.70–0.87)
	Source of control	Healthy control	0.79 (0.68–0.86)	0.82 (0.71–0.91)
		BPH/benign patients	0.75 (0.70–0.79)	0.77 (0.71–0.82)

Qualitative analysis	Overall			
	Ethnicity	Asian	0.32 (0.28–0.36)	0.99 (0.96–0.99)
		Other ethnicities	0.24 (0.22–0.27)	0.98 (0.96–0.99)
	Sample types	Serum	0.19 (0.16–0.21)	0.99 (0.98–0.99)
		Plasma	0.43 (0.32–0.56)	0.95 (0.87–0.99)
	Source of control	Healthy control	0.21 (0.19–0.27)	0.99 (0.97–1.00)
		BPH/benign patients	0.27 (0.25–0.29)	0.97 (0.96–0.99)
	Assay methods	N-MSP^*∗*^	0.39 (0.33–0.45)	0.99 (0.97–1.00)
		Other methods^&^	0.24 (0.22–0.26)	0.97 (0.96–0.98)
	Methylation gene location	GSTP1	0.41 (0.25–0.59)	0.98 (0.94–1.00)
		Other genes	0.22 (0.20–0.25)	0.98 (0.96–0.99)

^*∗*^MS-PCR (nonquantitative) and ^&^quantitative methylation-sensitive PCR; quantitative methylation-specific PCR; spectrophotometric assay; and bisulphite sequencing.
